# Cognitive-motor interventions based on virtual reality and instrumental activities of daily living (iADL): an overview

**DOI:** 10.3389/fnagi.2023.1191729

**Published:** 2023-06-15

**Authors:** Jorge Buele, Guillermo Palacios-Navarro

**Affiliations:** ^1^SISAu Research Group, Facultad de Ingeniería, Industria y Producción, Universidad Indoamérica, Ambato, Ecuador; ^2^Department of Electronic Engineering and Communications, University of Zaragoza, Teruel, Spain

**Keywords:** cognitive disorders, daily living, instrumental activities, motor intervention, virtual reality

## Abstract

Non-invasive, non-pharmacological interventions utilizing virtual reality (VR) represent a promising approach to enhancing cognitive function in patients with degenerative cognitive disorders. Traditional “pen and paper” therapies often lack the practical engagement in everyday activities that older individuals encounter in their environment. These activities pose both cognitive and motor challenges, underscoring the necessity of understanding the outcomes of such combined interventions. This review aimed to assess the advantages of VR applications that integrate cognitive-motor tasks, simulating instrumental activities of daily living (iADLs). We systematically searched five databases–Scopus, Web of Science, Springer Link, IEEE Xplore, and PubMed, from their inception until January 31, 2023. Our review revealed that motor movements, coupled with VR-based cognitive-motor interventions, activate specific brain areas and foster improvements in general cognition, executive function, attention, and memory. VR applications that meld cognitive-motor tasks and simulate iADLs can offer significant benefits to older adults. Enhanced cognitive and motor performance can promote increased independence in daily activities, thereby contributing to improved quality of life.

## 1. Introduction

Human life expectancy has seen a significant increase in recent decades (van Leeuwen et al., 2019). However, aging is accompanied by notable physical and cognitive changes that necessitate consideration (Elliott et al., 2021). Emerging technologies, including artificial intelligence, robotics, big data, telematics, and virtual and augmented reality, offer promising tools in health sciences to enhance individuals’ quality of life (Palacios-Navarro et al., 2016; Baig et al., 2019; Wildenbos et al., 2019; Hülür and Macdonald, 2020). VR systems have been leveraged for diagnosing age-related diseases and abnormalities in older adults (Varela-Aldás et al., 2022). Beyond diagnosis, VR has revolutionized therapeutic, training, and rehabilitation processes by providing immersive three-dimensional experiences aimed at restoring or maintaining a wide range of cognitive functions often compromised in older adults (Appel et al., 2020).

Executive functions (EF) are responsible for mental manipulation of information, concept formation, problem solving, and cue-directed behavior (Weintraub et al., 2012). They entail advanced cognitive skills such as working memory, inhibitory control, cognitive flexibility, planning, reasoning, and problem-solving (Cristofori et al., 2019). Reduced performance of these functions is quite evident when Mild Cognitive Impairment (MCI) is already present. Alzheimer’s disease (AD) in its early phase is particularly characterized by a diminished ability to mentally manipulate information (Elosúa et al., 2021). EF, related to working memory and attention, develop fully in adulthood and have direct links to cognitive efficiency, knowledge acquisition, academic performance, and autonomy (Baragash et al., 2022).

The reduction of autonomy generates problems in the person as it limits their ability to carry out their activities of daily living (ADL) (Tornero-Quiñones et al., 2020). EF interact with long-term memory in the episodic buffer, enabling the retrieval of previously learned information essential for problem-solving and new information processing for long-term storage (Oosterman et al., 2021). iADL demand greater effort from older adults, requiring continuous problem-solving related to their self-care (Cornelis et al., 2019). Key iADLs include meal preparation, budget planning, basic mathematical operations, and transportation use, necessitating active phonological loops to maintain auditory information in consciousness for immediate use (Weintraub et al., 2012). Patients with MCI or AD show significant impairments in long-term memory, EF, and spatial orientation (Murman, 2015).

Older adults often face physical limitations due to reduced motor and sensory system functionality (Khan et al., 2022), complicating both self-care activities (basic ADL or bADL) and mobility exercises such as walking, marching, or maintaining balance (Osoba et al., 2019). Research indicates that aerobic and balance physical training can increase muscle strength, thus reducing falls (Sherrington et al., 2019). It is important to note that while young and cognitively healthy adults generally do not exhibit postural and gait control problems, older adults or those with cognitive issues are vulnerable to cognitive distractions (dual or additional tasks) that may compromise postural control (Zhang et al., 2019). Studies like that of Sato (2017) have shown that EF partly governs the motor and sensory system, meaning that its malfunctioning is linked to falls. These findings underscore the need for cognitive interventions that also incorporate physical exercises, aiming for a comprehensive approach (Tromp et al., 2015).

Conventional cognitive therapies, or “paper and pencil” therapies, have long been used to treat cognitive impairments in older adults. These cost-effective, easily accessible, and clinically validated therapies include tabletop activities like puzzles, wooden blocks, card games, and mazes (Bernini et al., 2019). However, these therapies often struggle to assess patients’ cognitive levels systematically and keep them engaged. Computer-based cognitive interventions, on the other hand, have emerged as a promising alternative, demonstrating effectiveness in improving cognitive function in both healthy older adults and those with neuropsychological disorders (Thapa et al., 2020; Zuschnegg et al., 2023). Unlike conventional therapies, these computer-based interventions are capable of systematically adjusting task difficulty according to the individual’s cognitive level, offering a more diverse and engaging range of programs and activities (Wollesen et al., 2020). These interventions surpass static and straightforward training, providing interactive and immersive experiences.

While computer-based cognitive interventions offer many advantages, it is crucial to establish specific guidelines to assist healthcare professionals in determining the appropriate activities and clinical conditions for implementation (Goldstein and McNeil, 2012). Standardizing these guidelines can enhance the effectiveness and efficiency of computer-based interventions in clinical practice. Given the existing digital divide between younger and older adults, it’s vital to consider how this might impact older individuals’ access to modern technologies like VR. VR-based cognitive interventions, leveraging advancements in information technology, have shown great promise (Liao et al., 2020). While some older adults have successfully used these technologies for social interaction and cognitive enhancement (Gao et al., 2020), others may face social exclusion due to a lack of necessary skills and equipment. Addressing this digital divide is essential to ensure older individuals can fully benefit from VR/AR-based interventions for cognitive enhancement and overall wellbeing.

Research shows that VR can significantly enhance cognitive-motor interventions in older adults (Kwan et al., 2021). For instance, the study of Pichierri et al. (2012) incorporated traditional physical exercise and dance video games into cognitive-motor training. This dual-task exercise led to VR system users taking quicker steps than the passive control group, potentially preventing falls in real-life situations. Another study (Torpil et al., 2021) featured various serious games, including activities such as a boxing trainer, a running game controlled by jumping and body movements, a penalty kick game, and a skydiving game controlled by body and shoulder movements. The results showed improvements in visuospatial perception, organization, orientation, attention, and concentration compared to the control group. The study of Cameirão et al. (2016) demonstrated how mood and cognitive functioning can supplement physical rehabilitation, as post-stroke participants had to locate a target image within a set of 15 distractors for attention and memory training.

Several reviews have analyzed VR’s effect on cognitive and motor functions. A systematic review conducted by Zhu et al. (2021) included 11 randomized controlled trials (RCTs), revealing a moderate impact of VR interventions on cognitive and motor function, including attention/execution, memory, global cognition, and balance in patients with mild cognitive impairment (MCI) and dementia. Gao et al. (2021) analyzed six VR interventions combined with traditional rehabilitation, showing significant improvements in general cognition, attention, and mood, though not in global cognition, motor function, and ADLs. In the review of Pichierri et al. (2011) computerized interventions (mostly VR) showed positive effects on various physical abilities in older adults with TBI and stroke compared to non-VR proposals. Participants displayed greater motivation and compliance with the computerized environment compared to regular physical training programs. A similar approach was used in the study of Schoene et al. (2014), where the use of technological tools in cognitive-motor rehabilitation was analyzed. The two studies that used a VR environment with a treadmill reported improvements in balance and mobility.

The benefits of VR in training older adults are evident based on the existing evidence. However, the heterogeneity of activities simulated in VR environments makes analysis challenging. Therefore, this review focuses solely on VR applications in which participants perform iADLs and motor tasks (iADL-m) to assess their impact on the cognitive functions of older adults.

## 2. Methods

### 2.1. Eligibility criteria

This review adheres to the PRISMA (Preferred Reporting Items for Systematic reviews and Meta-Analyses) guidelines for systematic reviews. Inclusion criteria encompass: (i) articles published in English; (ii) studies without temporal or spatial restrictions; (iii) interventions employing VR-iADL and motor applications; (iv) clinical trials and pilot studies; (v) studies involving healthy older adults with MCI or dementia; (vi) immersive and non-immersive systems; and (vii) studies both with and without a control group.

### 2.2. Exclusion criteria

Exclusion criteria include: (i) theoretical or descriptive studies; (ii) non-peer-reviewed articles and preprints; (iii) studies not employing VR; (iv) applications that do not simulate an iADL; (v) interventions solely cognitive in nature; (vi) literature reviews; (vii) interventions for disorders other than those specified; (viii) diagnostic or screening investigations; and (ix) interventions without a pretest and posttest.

### 2.3. Data sources and search strategy

Relevant articles were identified in Pubmed, Scopus, IEEE Xplore, Apa PsycNet, and the Web of Science databases, from their inception until January 2023. The search terms included keywords relating to virtual reality (e.g., “virtual,” “computer”), cognitive-motor interventions (e.g., “cognitive,” “motor,” “memory,” “executive,” “rehabilitation,” “training,” “stimulation”), iADL (e.g., “daily,” “ADL,” “iADL,” “store,” “shopping,” “supermarket,” “cook,” “cooking,” “kitchen”), and cognitive disorders [e.g., “mild,” “Alzheimer,” “dementia,” NOT (“stroke,” “brain injury,” “TBI”)].

### 2.4. Study selection

Search terms were tailored to each database. The titles and abstracts of articles in each database were screened independently by two authors (JB and GP-N) in line with the specified inclusion and exclusion criteria. Duplicate studies were removed, and additional studies cited in the identified articles were included. The selected articles were then stored, organized, and assessed using the Mendeley bibliographic manager v1.19.8 (Mendeley Ltd., Elsevier, Netherlands).

## 3. Results

Upon identifying articles that met the inclusion and exclusion criteria, data were extracted from each article. This included the authors, the study design, the sample, the motor and cognitive intervention applied, and the main findings. Table 1 presents the characteristics of each study.

**TABLE 1:** Characteristics of the included studies.

References	Design	Sample	VR intervention motor	Cognitive	Major findings
Liao et al., 2020, Taiwan	RCT	34 MCI patients (23F/11M)	Aerobic and resistance exercises, cleaning windows, tai chi, walking, bending and lifting objects at home	Shopping, preparing food, managing finances and transportation	Decreased activation in prefrontal areas indicating increased neural efficiency. Dual VR tasks (physical and cognitive) could have positive effects on several cognitive functions
Liao et al., 2019, Taiwan	RCT	34 MCI patients (23F/11M)	Aerobic and resistance exercises, walking, getting on and off a stool	Shopping, preparing food, managing finances and transportation	The training improved divided attention (cognitive and motor at the same time) and cognitive flexibility (aspects of EF). VR was able to increase motivation
Park et al., 2020, Republic of Korea	RCT	35 MCI patients (17F/18M)	Moto Cog: personal hygiene, driving, door opening, shampoo, use of handles	Card game, puzzle, construction activity with sticks and mazes	The VR group improved in the MoCA, TMT and DST tests performance. Motivation improved with positive effects on attention and short-term visuospatial memory
Doniger et al., 2018, Israel	RCT	34 healthy older adults with a history of Alzheimer’s disease	Walking on a treadmill	View a virtual shopping list and pick up items, plan purchases with 100 shekels (Israel currency)	Shopping tasks are very important to maintain and/or regain independence. There was a neuroplastic change in DTI measures of the hippocampus in just 2 h of training
Mrakic-Sposta et al., 2018, Italy	Pilot Study	10 MCI and MD patients (6F/4M)	Cycling in a park, cross the road by bike avoiding cars	Virtual shopping: List of five items, choose the aisle and the product	Improvements in the MMSE, ROCFT, FAB and AM tests performance. Increased antioxidant capacity and reduction of lipid peroxidation and DNA damage. Better performance in real life thanks to self-perceived improvement and motivation
Arlati et al., 2017, Italy	Pilot Study	10 patients with MCI	Cycling in nature, cross and avoid cars by bike	buy five items	Patients showed better performance in real life (less forgetfulness). The trick words (distractors) did not change the results. Patients exhibited high levels of commitment and motivation
Kwan et al., 2021, China	RCT	17 MCI patients (15F/2M)	Bicycle exercise on an ergometer	Shopping, transportation, cooking, bird watching, reporting lost items	Patients reported better performance in real life because of self-perceived improvements. The intervention could improve cognition and frailty, reducing the risk of falls, disability and mortality.
McDaniel et al., 2014, United States	RCT	96 healthy older adults (61F/35M)	Setting the table with cutlery, flexibility exercises (aerobics)	Prepare breakfast: five foods, board game and remember health facts	Participants’ performance did not improve in the kitchen task. Aerobic exercises did not show significant changes. Cognitive training did not transfer to other tasks.

AM, attentional matrices test; DST, digit span test (forward/backward); DTI, diffusion tensor imaging; F, female; FAB, frontal assessment battery; M, male; MMSE, mini-mental state examination; MoCA, montreal cognitive assessment; Moto cog, VR training program focused on improving upper extremity ADL performance; ROCFT, Rey-Osterrieth complex figure test; TMT, trail making test (A/B).

## 4. Discussion

With advancing age, the ability to perform motor-related activities becomes increasingly challenging. As noted by Muir et al. (2012), elderly individuals with MCI demonstrate a reduction in gait speed and an elongated stride completion time when transitioning from a single task to a double one. Older adults with cognitive difficulties are at a higher risk of falls compared to their healthy counterparts, and this risk escalates when the walking speed drops below 1 m/s (Abellan Van Kan et al., 2009). Furthermore, elderly people with MCI are likely to make risky decisions when crossing streets (Rizzo et al., 1997). An increase in latency at the onset of a journey and a slower pace are also reported (Bahureksa et al., 2016), indicating a correlation between gait issues and the onset of Alzheimer’s Disease (AD) due to associated visuospatial deficits (Fukui and Lee, 2009; Rosso et al., 2019). A reduction in walking speed is perceived as an indicator of cognitive frailty that impacts wellbeing and survival (Duan-Porter et al., 2019; Van Schooten et al., 2019; Beltz et al., 2022). From a cognitive standpoint, memory and executive functions (EF) are most frequently impacted by AD.

### 4.1. Benefits in cognitive functions

Cognitive-motor interventions have demonstrated potential as an effective method to boost cognitive functions and alleviate frailty. Evidence indicates that these interventions can enhance physical performance, brain functionality (as determined by resting functional magnetic resonance imaging), and cognitive capacities (Raichlen et al., 2020; Yue et al., 2020). The use of VR for such interventions has been successfully demonstrated in both healthy elderly individuals and those suffering from dementia (Yi et al., 2022).

A recent review highlighted that VR cognitive training can exert moderate to large effects on global cognition, attention, memory, and motor performance in individuals with Mild Cognitive Impairment (MCI), with additional benefits for executive function seen in people with dementia (Papaioannou et al., 2022). The VR-integrated ADL motor studies explored in our review have successfully motivated individuals to utilize learned skills in real life, with encouraging knowledge transfer outcomes (McDaniel et al., 2014). Several studies (Healy et al., 2005; Dahlin et al., 2008) propose that transferable skills are only developed through practice, suggesting that other non-iADL applications may not yield the same benefits. More specifically, evidence suggests that practicing an activity can facilitate the transfer of coordination skills in dual tasks, as demonstrated in the study by Schubert et al. (2017). The review conducted by Joubert and Chainay (2018) indicated a slight superiority for combined physical and cognitive training over training conducted separately. However, separate training also influences different cognitive functions, warranting further research. The links between cognitive and motor processes are not new, and they likely share a similar evolutionary history (Leisman et al., 2016).

The study carried out by Liao et al. (2020) reported that the stimulation of dual tasks (physical and cognitive) simulating ADL in virtual environments can impact various cognitive functions, notably executive functions (EF) and memory (Lauenroth et al., 2016). In previous work, the authors (Liao et al., 2019) created a VR application simulating iADL-m, which aided in training divided attention and cognitive flexibility (aspects of EF), targeting individuals to achieve between 50 and 75% of their maximum heart rate. Concurrently, the results of the study of Mrakic-Sposta et al. (2018) revealed improvements in attention, EF, and memory in patients with MCI. Similar improvements in EF and walking speed were also observed in Kwan et al. (2021)’s study, consistent with a recent review (Papaioannou et al., 2022). These applications provide real-time feedback, leading to long-term benefits, such as the transfer of knowledge to the real world (Ross et al., 2016).

McDaniel et al. (2014) combined virtual food preparation, table setting, and flexibility exercises, showing benefits for prospective memory. This involves spontaneous recovery and care processes that enable defining the actions to be carried out based on the place and situation. High levels of physical activity correlated directly with the proper functioning of EF, suggesting a potential palliative measure (Galle et al., 2022). The study of Galle et al. (2023) showed that those who increased their physical activity by more than 30% displayed improvements in gait speed, aerobic capacity, EF, and global cognition compared to those who did not. Significant enhancements in spatial cognition were also reported, implying that orientation practice could potentially forestall the cognitive decline of the elderly. Applications simulating a virtual city and requiring street crossings could be beneficial to users (Waddington and Heisz, 2023).

According to the study of Doniger et al. (2018), dual processing speed (mobility) training offered protection against dementia. As demonstrated in the longitudinal study carried out by Edwards et al. (2016) cognitive-motor training reduced the experimental group’s chances of developing dementia after 10 years by 33%. Although this study primarily aimed to improve cognitive function with simultaneous walking being an incidental action inherent to the shopping task, the results indicated that cognitive training benefited physical performance (PE) while physical training benefited memory. The study of Kwan et al. (2021) reported improvements in cognitive function as a result of virtual cognitive-motor training, which can be attributed to neural plasticity. This effect could be due to the super additive synergistic effects created by the multitasking requirement of simultaneous physical and cognitive exercises. This aligns with the findings of Herold et al. (2018), who suggested that motor training incorporating a cognitive task has the highest ecological validity. Interventions that encourage significant physical exercise, offer variable levels of difficulty, and maintain a task-focused approach have shown to be more effective in adapting to related tasks (Stanmore et al., 2019; Wollesen et al., 2020). This suggests that a comprehensive, multifaceted approach can be more beneficial for cognitive health and physical function.

### 4.2. Changes in brain function

Numerous research studies have underscored the advantages of physical exercise as a complementary approach in cognitive rehabilitation. Liao et al. (2020) reported that such exercise promotes the release of brain-derived neurotrophic factor, enhancing blood flow and exerting beneficial metabolic effects. Park et al. (2020) further emphasized this in a virtual cognitive-motor intervention study, indicating that physical exercise stimulates the hypothalamic-pituitary-adrenal axis, thereby increasing cortisol levels and enhancing learning and memory (Luger et al., 1987). Doniger et al. (2018) observed that groups engaged in cognitive-motor training demonstrated increased cerebral blood flow in the prefrontal, middle, and posterior cingulate cortices. This could be indicative of heightened brain activity, even though the motor task was mild, potentially sparking neuroplastic changes given the brain’s cognitive reserve (Esiri and Chance, 2012; Chapman et al., 2015). This hypothesis aligns with animal studies demonstrating new neuronal and synaptic connections in advanced age, increased cortical thickness, enhanced brain weight, and changes in blood flow (Mora, 2013). Contrastingly, groups engaged solely in motor exercise exhibited changes in the hippocampus without modifications in cerebrovascular reactivity (Chapman et al., 2016).

Theories of brain plasticity have been at the forefront of recent research (Chiu et al., 2017; Yamada and Sumiyoshi, 2021). The combination of increased cerebral blood flow and its synergistic response on global cognitive function could foster the nervous system’s ability to reorganize neuronal activity and function, a process known as neuroplasticity, thereby enhancing cognitive learning (Kwan et al., 2021). Diminished activation of prefrontal areas is linked with greater neural efficiency, while reduced activation of frontoparietal areas suggests improved cognitive performance post-training (Schättin et al., 2016; Vermeij et al., 2017). In a similar vein, Park et al. (2020) asserted that cognitive-motor interventions stimulate brain neurotransmitters, particularly the cholinergic and dopaminergic systems, thereby bolstering concentration and memory in older adults (Hwang et al., 2021; Yang et al., 2022). The literature consistently highlights the impact of physical exercise on cognitive functions, particularly executive functions, due to the release of brain-derived neurotrophic factor and increased hippocampal blood flow, both of which result in favorable metabolic effects (Liao et al., 2020).

### 4.3. Implications for practice

Traditional cognitive tests, primarily pencil-and-paper based, have received criticism for their inherent limitations, including the omission of key factors such as an individual’s education level (Kessels, 2019; Palacios-Navarro et al., 2022). However, the emergence of VR and ecological momentary assessments through wearable devices present innovative alternatives capable of enhancing precision and sensitivity in cognitive assessments (Chan et al., 2018; Hartle et al., 2021). These methods not only supplement traditional tests but also pave the way for a new direction in rehabilitation interventions. The landscape of cognitive treatment methodologies is evolving, with both traditional and VR-based techniques demonstrating efficacy. Both conventional cognitive training programs and VR-based interventions have induced improvements in cognition and executive functions among older adults. Specifically, VR interventions shine in their capacity to simulate ADLs, thereby emerging as a promising tool for cognitive rehabilitation in early stage cognitive disorders (Wollesen et al., 2020; Matsangidou et al., 2023).

Immersive VR offers a stimulating and engaging experience for seniors, enhancing their motivation and enjoyment. Besides its adaptability and high level of automation, these features can alleviate the workload for caregivers and medical professionals (Bauer and Andringa, 2020). Immersive VR also fosters a sense of autonomy in older adults, which is vital for their emotional and cognitive wellbeing. The current review and recent studies suggest that older adults can tolerate and significantly benefit from immersive VR regarding cognitive and physical health (Yi et al., 2022; Matsangidou et al., 2023), as highlighted by Słyk et al. (2019) in their systematic review. However, it is of utmost importance to tailor these interventions to the individual needs and capabilities of participants, necessitating adjustments in task difficulty, session duration, and supervision during training.

Virtual reality, particularly when simulating iADLs, is carving a niche in cognitive-motor rehabilitation (Arlati et al., 2017). Such training programs are well received by patients and can be applied across various clinical environments, including community centers and senior care facilities (Kwan et al., 2021). Patient safety is paramount, requiring careful evaluation before inclusion in these programs and vigilant monitoring for symptoms of VR-induced dizziness post-training (Kwan et al., 2021). VR exhibits significant flexibility in its implementation, suitable for a range of clinical contexts from neurology clinics and rehabilitation centers to home care settings (McDaniel et al., 2014). Successful implementation, however, hinges on the availability of appropriate equipment and trained personnel. In conclusion, VR holds immense potential to revolutionize the treatment of cognitive disorders, offering a personalized and motivating approach to rehabilitation.

### 4.4. Future implications

When designing future studies that utilize iADL-m based VR interventions, it is imperative to consider the sociodemographic characteristics of participants. Factors such as age, education level, family history, and cognitive disorders should be accounted for, as these variables could potentially influence the results. Moreover, interventions need to be tailored to each participant’s physical condition to avoid injury or loss of motivation. To this end, maintaining a heart rate between 50 and 75% of the maximum is suggested, which some may perceive as moderately intense. Notably, most participants in the reviewed studies were primary-educated women, highlighting the need to consider these variables when designing effective interventions for older adults.

The duration of interventions also requires careful consideration. The systematic review conducted by Kelly et al. (2014) evaluated training and cognitive stimulation in older adults and their influence on cognition and daily functioning. They identified that studies lasting at least 10 sessions and whose exercises are adapted to the conditions of the population using level advancements or hints have higher skill maintenance effects. It is important to consider that a high number of sessions could generate a repeated learning effect that promotes disinterest and unreliable results, as mentioned by Cooley et al. (2015). Furthermore, McDaniel et al. (2014) suggested that, although aerobic exercise can enhance certain tasks, repeating the same exercise for an extended period might decrease interest and motivation.

In terms of experimental design aimed at improving cognitive and physical health, the inclusion of an active control group is recommended for more accurate comparisons. It is also important for participants to follow similar training routines, either through comparable interactive applications or by conducting physical and cognitive exercises at home using accessible interactive headsets. Ensuring that the application is specially designed for the study population and adapts the difficulty levels to each individual’s characteristics is crucial. Strategies such as incorporating clues or positive feedback in the systems, as suggested by Arlati et al. (2017), can help maintain participants’ motivation. In the same vein, including distractors, like presenting several brands of the same product in a simulated supermarket task, can test the participants’ concentration. However, it is essential to consistently use and position the same elements to prevent information bias (Arlati et al., 2017).

As mentioned by Wollesen et al. (2020), traditional interventions that combine cognitive and motor aspects, along with technology-based exercise games, have shown favorable impacts on general cognition and inhibitory capacity in older adults. However, due to the heterogeneity of studies concerning interventions, measurements, and results, caution is needed when interpreting these results. Future research may explore the benefits and challenges of a hybrid approach that combines “pencil and paper” activities with an exercise program, enhanced by interactive technology. Some proposals have suggested a training program that merges both real and virtual environments (Foloppe et al., 2018). This approach could potentially be replicated in a more diverse and representative sample. These integrated approaches have the potential to optimize cognitive and physical outcomes in older adults by leveraging the benefits of conventional therapies–which are accessible, affordable, easy to use, and clinically validated–and the personalization and motivation offered by interactive-based interventions.

By considering the unique needs and capabilities of older individuals, comprehensive interventions that combine physical and cognitive exercises can be developed. This holistic approach optimizes the potential for cognitive function improvement, promotes independence, and enhances older adults’ overall quality of life. In particular, new technologies can supplement unsupervised home training methods, providing older individuals with a handy tool for conducting appropriate training sessions independently. By offering immersive and stimulating experiences, interactive technology can boost older adults’ motivation and engagement, which may, in turn, positively impact their training outcomes and quality of life.

## 5. Conclusion

This review provides compelling evidence that iADL-based VR cognitive-motor interventions can notably enhance both cognitive function and motor skills in individuals with Mild Cognitive Impairment (MCI) and dementia. These interventions hold significant promise, particularly in enhancing independence, functional ability in ADL, cognitive functions, and reducing frailty among older adults. Moreover, these VR-based interventions potentially offer a more engaging and motivational alternative to conventional therapies, which could improve treatment adherence and outcomes.

The literature suggests that the relationship between cognitive exercise and motor functions is bidirectional–cognitive training can positively impact motor performance, and conversely, motor training can have beneficial effects on cognitive function. Moreover, practicing specific activities can enhance coordination skills in dual tasks, contributing to the training of divided attention and cognitive flexibility. Thus, VR cognitive-motor interventions centered on iADLs can effectively aid in the transfer of acquired skills to the performance of daily activities. However, the choice of the appropriate intervention should be tailored to each individual, considering their needs, personal goals, and physical and motor conditions. Combining physical and cognitive training may yield slightly better results than training these areas separately. Nonetheless, further research is needed to deepen our understanding of how each training type affects different cognitive domains.

## Author contributions

JB and GP-N contributed to conception, design of the study, and wrote the first draft of the manuscript. JB organized the database. Both authors contributed to manuscript revision, read, and approved the submitted version.
